# Model‐Derived Iopamidol Clearance and Creatinine‐Based Kidney Function Estimates After Trauma: A Retrospective Cohort Study

**DOI:** 10.1002/phar.70219

**Published:** 2026-11-09

**Authors:** Manjunath P. Pai, Aleksas Matvekas, Lu Wang, Levi T. Hooper, June Sullivan, Raymond Jean, Kathleen A. Stringer

**Affiliations:** ^1^ Department of Clinical Pharmacy, College of Pharmacy University of Michigan Ann Arbor Michigan USA; ^2^ Department of Surgery, Michigan Medicine University of Michigan Ann Arbor Michigan USA; ^3^ Max Harry Weil Institute for Critical Care Research and Innovation University of Michigan Ann Arbor Michigan USA; ^4^ Division of Pulmonary and Critical Care Medicine University of Michigan Ann Arbor Michigan USA

**Keywords:** contrast media, creatinine, drug dosage calculations, glomerular filtration rate, kidney function tests, multiple trauma

## Abstract

**Study Objective:**

To determine whether iopamidol administered during clinically indicated contrast‐enhanced computed tomography (CE‐CT) and measured in remnant serum can provide an individualized surrogate value for measured glomerular filtration rate (mGFR) after trauma to compare against creatinine‐based estimates.

**Design:**

Single‐center retrospective observational cohort.

**Setting:**

Academic medical center.

**Patients:**

Seventy‐six adults evaluated for trauma who underwent iopamidol‐enhanced CT and had at least one quantifiable postcontrast concentration and serum creatinine measurement.

**Intervention:**

None.

**Measurements and Main Results:**

Population parameters of a previously developed two‐compartment structural model were re‐estimated using the study data and stochastic approximation expectation–maximization, followed by participant‐specific conditional‐mode clearance estimation. Individual iopamidol clearance was interpreted as mGFR (mGFR_IOP_). Agreement with Cockcroft–Gault creatinine clearance and nonindexed Chronic Kidney Disease Epidemiology Collaboration (CKD‐EPI) creatinine estimated GFR was assessed; 449 serum creatinine measurements were modeled over 7 days. Median mGFR_IOP_ was 99 mL/min (interquartile range, 59–125). Each 30‐mL/min higher mGFR_IOP_ was associated with a 0.11‐mg/dL lower serum creatinine at iopamidol administration (95% confidence interval, 0.06–0.16; *p* < 0.001). Predicted serum creatinine values converged over 7 days despite differences in mGFR_IOP_ (interaction *p* < 0.001). Estimates were within 30% of mGFR_IOP_ in 71% of patients for CKD‐EPI versus 59% for Cockcroft–Gault (*p* = 0.035). CKD‐EPI had narrower 95% limits of agreement compared with Cockcroft‐Gault (−73 to 57 vs. −82 to 74 mL/min, respectively) but increasingly underestimated higher mGFR_IOP_ (proportional‐bias *p* < 0.001).

**Conclusion:**

Cohort‐specific iopamidol clearance estimates as a surrogate for mGFR can be generated from routine CE‐CT and remnant serum samples that differ from creatinine‐based estimates. Prospective validation against conventional mGFR is required before clinical use.

## Graphical abstract

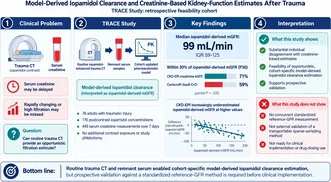

## 1 Introduction

Kidney function can change rapidly after major trauma because of shock, resuscitation, systemic inflammation, and nephrotoxic therapies. Both acute kidney injury (AKI) and augmented kidney clearance can influence fluid management, medication exposure, and prognosis [1, 2]. Clinical surveillance relies largely on serum creatinine (S_Cr_) and urine output. S_Cr_ is a delayed functional marker that may be obscured by fluid resuscitation, altered creatinine generation, and an unknown preinjury baseline. Creatinine‐based equations are also unreliable when glomerular filtration rate (GFR) is changing or supranormal [2, 3, 4].

Contrast‐enhanced computed tomography (CE‐CT) is routinely performed during trauma evaluation and provides a precisely timed intravenous dose of an exogenous filtration marker. Unlike endogenous biomarkers, clearance of an administered marker directly reflects its elimination from plasma. We previously demonstrated that iopamidol and iohexol clearance provide comparable measures of the GFR, supporting iopamidol‐derived measured GFR (mGFR_IOP_) alongside iohexol, the reference marker used to validate contemporary estimating equations [5, 6]. Iopamidol already administered during clinical care, combined with remnant blood specimens, may therefore provide an opportunistic filtration phenotype without additional contrast exposure or phlebotomy. The primary objective was to compare creatinine‐based kidney‐function estimates with model‐derived iopamidol clearance, interpreted as mGFR_IOP_. As an exploratory secondary objective, we examined the association between mGFR_IOP_ at imaging and subsequent S_Cr_ trajectories [6].

## 2 Methods

### 2.1 Study Design and Participants

This study was approved by the University of Michigan Medical School Institutional Review Board (HUM00275396; “Trauma Renal Function Assessment by Contrast Enhancement [TRACE Study]”; June 13, 2025). The requirement for study‐specific informed consent was waived. Adults aged 18 years or older who were evaluated for traumatic injury, received iopamidol‐enhanced CT between September 2025 and March 2026, and had at least one remnant clinical serum specimens collected within 72 h were eligible for specimen retrieval. Participants were excluded if specimens were unavailable, no postcontrast iopamidol concentration was quantifiable, individual clearance could not be estimated, or no S_Cr_ measurement was available. No outcome was prespecified because the study was exploratory. However, once analysis was complete, the primary outcome was agreement of Cockcroft–Gault (using actual weight) creatinine clearance (CrCl) and creatinine‐based nonindexed Chronic Kidney Disease Epidemiology Collaboration (CKD‐EPI) estimated GFR (eGFR_cr_) with mGFR_IOP_, summarized by P30. P30 was defined as the proportion of participants whose creatinine‐based estimate was within 30% of iopamidol‐derived mGFR. Secondary agreement measures included bias, absolute error, root mean squared error, and Bland–Altman limits of agreement. The association between iopamidol‐derived mGFR and longitudinal S_Cr_ was a secondary outcome. This study is reported in accordance with the STROBE recommendations. Cohort assembly and data‐quality procedures are detailed in the Supporting Information. Detailed measures of trauma severity and potential determinants of subsequent S_Cr_, including Intensive Care Unit (ICU) admission, bleeding or transfusion, fluid balance, vasopressor exposure, and nephrotoxin use, were not systematically collected under this feasibility protocol.

### 2.2 Iopamidol Measurement and Pharmacokinetic Analysis

Remnant serum specimens were stored at −70°C, and iopamidol concentrations were quantified using a previously described liquid chromatography–mass spectrometry method [6]. Administered mass was calculated from documented contrast volume and formulation. Analyses were performed in MonolixSuite 2024R1 (Lixoft SAS, Antony, France). Each participant's complete dosing and concentration‐time history was analyzed, with power‐injector administration represented as an intravenous bolus. The previously published two‐compartment iopamidol model supplied the structural framework and initial parameter values [6]. Population fixed effects, interindividual variability, and residual‐error parameters were re‐estimated from the concentration‐time data using stochastic approximation expectation–maximization; published population parameters were not held fixed. Participant‐specific conditional‐mode clearance estimates were subsequently calculated from the cohort‐updated population model. Individual iopamidol clearance was converted to mL/min and interpreted as absolute, nonindexed mGFR_IOP_. S_Cr_, eGFR_cr_, and CrCl were excluded as clearance covariates [7, 8]. Bioanalysis, model specification, estimation, diagnostics, and sensitivity analyses are provided in the Supporting Information.

### 2.3 Statistical Analysis

Agreement of mGFR_IOP_ with CrCl and eGFR_cr_ was assessed using bias, absolute error, root mean squared error, P30, and Bland–Altman analysis. Bias was defined as the creatinine‐based estimate minus mGFR_IOP_ [7, 8]. Differences in paired P30 values were evaluated using an exact McNemar test. As an exploratory secondary analysis, the association between index iopamidol‐derived mGFR and subsequent S_Cr_ was evaluated using a mixed‐effects model with participant‐specific random intercepts, flexible time effects, and an mGFR_IOP_‐by‐time interaction. This descriptive analysis was not intended to establish a causal effect of baseline mGFR_IOP_ or to adjudicate subsequent AKI. Complete model specifications are provided in the Supporting Information. All available S_Cr_ measurements were aligned to time from the index iopamidol administration without imputing an unobserved pre‐trauma baseline. Continuous variables were summarized as median (interquartile range [IQR]) and categorical variables as number (percentage). Complete statistical methods are provided in the Supporting Information.

## 3 Results

Identification and selection of the analytic study cohort is illustrated in Figure S1 with more details in the Supporting Information. Of 90 patients reviewed, 84 met eligibility criteria and specimens were retrievable for 80 patients. Four patients were excluded because no retrieved specimen contained quantifiable postcontrast iopamidol, leaving 76 participants. Table 1 summarizes the final cohort (*n* = 76). Median age was 60 years (IQR, 44–74 years), 63.2% were male, and median body mass index was 28.8 kg/m^2^ (IQR, 24.8–32.5 kg/m^2^). The initial eGFR_cr_ was less than 60 mL/min/1.73 m^2^ in 34.2% and at least 90 mL/min/1.73 m^2^ in 25.0%. Participants received a median initial iopamidol dose of 76.5 g (IQR, 75.4–76.5 g). Individual mGFR_IOP_ was estimated from 176 concentrations, with a median of 2 (IQR, 1–3) per participant; model results are reported in Table S1.

**TABLE 1:** Demographic, clinical, kidney‐function, and sampling characteristics.

Characteristic	Study cohort (*N* = 76)
Age, years	60 [44–74]
Male sex	48 (63.2%)
Weight, kg	84.7 [69.6–103.2]
Height, cm	172.7 [164.0–180.3]
Body mass index, kg/m^2^	28.8 [24.8–32.5]
Body mass index ≥ 30 kg/m^2^	32 (42.1%)
Body surface area, m^2^	2.00 [1.80–2.28]
First available S_Cr_, mg/dL	1.11 [0.96–1.33]
Cockcroft‐Gault CrCl, mL/min	88.5 [54.9–114.9]
CKD‐EPI 2021 eGFR, mL/min/1.73 m^2^	74.5 [53.4–89.9]
Nonindexed CKD‐EPI eGFR, mL/min	89.9 [60.7–107.4]
eGFR < 30 mL/min/1.73 m^2^	2 (2.6%)
eGFR 30–59 mL/min/1.73 m^2^	24 (31.6%)
eGFR 60–89 mL/min/1.73 m^2^	31 (40.8%)
eGFR ≥ 90 mL/min/1.73 m^2^	19 (25.0%)
Initial iopamidol dose, g	76.5 [75.4–76.5]
Iopamidol concentrations per participant	2 [1–3]
S_Cr_ measurements per participant	5.5 [3–7]

*Note:* Data are median [IQR] or *n* (%).

Abbreviations: CKD‐EPI, Chronic Kidney Disease Epidemiology Collaboration; CrCl, creatinine clearance; eGFR, estimated glomerular filtration rate; S_Cr_, serum creatinine.

Median mGFR_IOP_ was 99 mL/min (IQR, 59–125 mL/min). CrCl had a mean bias of −4.0 mL/min, P30 of 59.2%, and 95% limits of agreement from −82 to 74 mL/min (Figure 1A). The mean bias and P30 values were lowest with actual body weight rather than any other evaluated body size descriptor used for CrCl. Nonindexed eGFR_cr_ had higher P30 (71%; paired *p* = 0.035) and narrower limits of agreement (−73 to 57 mL/min) but increasingly underestimated kidney function as mGFR increased (*p* < 0.001 for proportional bias; Figure 1B). The direction of these comparisons was unchanged among participants with at least 2 or at least 3 concentrations and among those with a single verified dose. Complete agreement and sensitivity results are provided in Tables S2 and S3.

**FIGURE 1:**
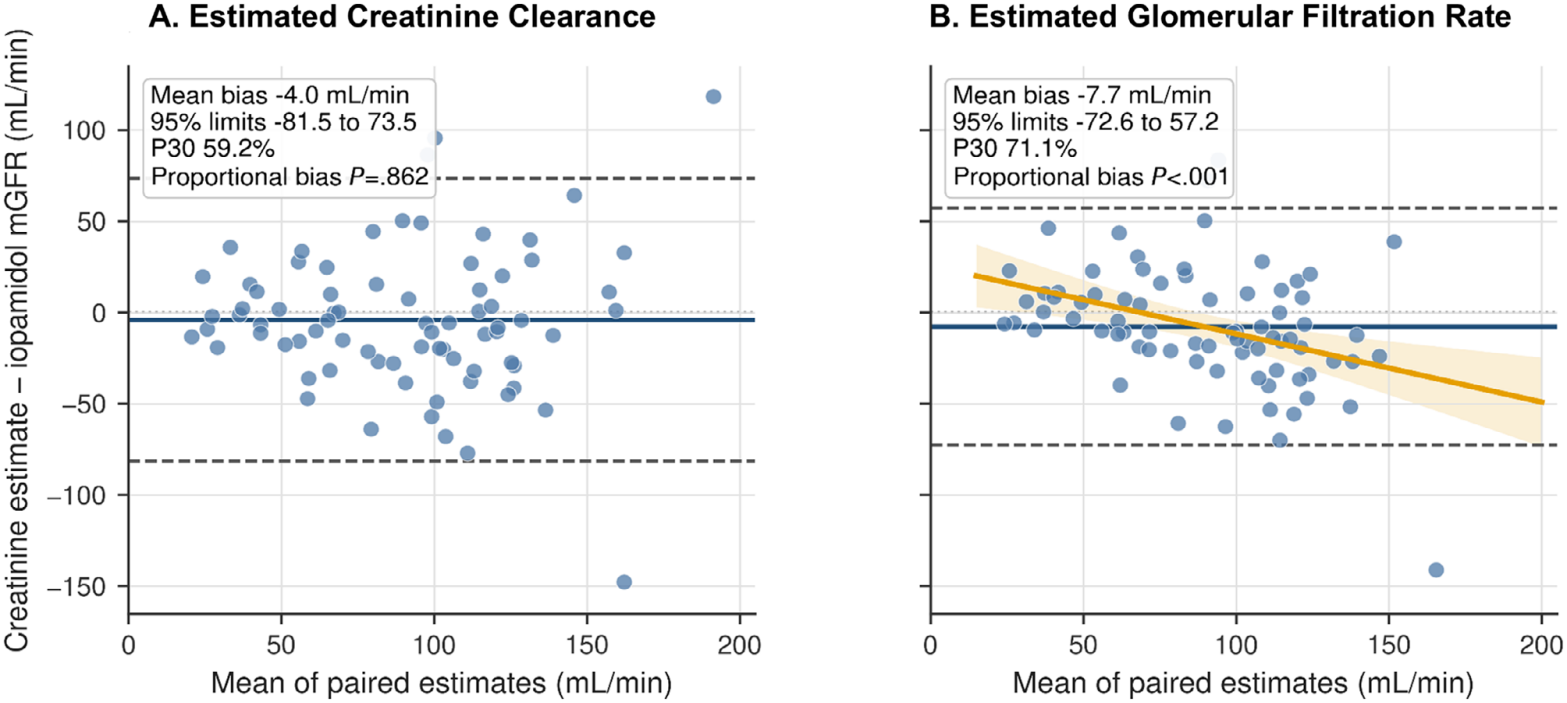
Relationship of iopamidol‐derived measured glomerular filtration rate with creatinine‐based kidney‐function estimates. Bland–Altman agreement of iopamidol‐derived measured Glomerular Filtration Rate (mGFR) with Cockcroft–Gault (actual body weight) creatinine clearance (A) and nonindexed 2021 Chronic Kidney Disease Epidemiology (CKD‐EPI) creatinine estimated Glomerular Filtration Rate (eGFR) (B), respectively. Differences were calculated as the creatinine‐based estimate minus iopamidol‐derived mGFR. Solid horizontal lines indicate mean bias, and dashed lines indicate the 95% limits of agreement. The fitted trend line in panel B demonstrates increasing underestimation by CKD‐EPI at higher kidney function.

The longitudinal analysis included 449 S_Cr_ measurements, with a median of 5.5 (IQR, 3–7) per participant. Observed S_Cr_ varied substantially within and among participants (Figure 2A). At iopamidol administration, each 30‐mL/min higher mGFR_IOP_ was associated with a 0.11‐mg/dL lower S_Cr_ (95% CI, 0.06–0.16 mg/dL lower; *p* < 0.001). The mGFR_IOP_‐by‐time interaction was significant (*p* < 0.001), indicating that the association between index mGFR_IOP_ and predicted S_Cr_ attenuated over 7 days (Figure 2B).

**FIGURE 2:**
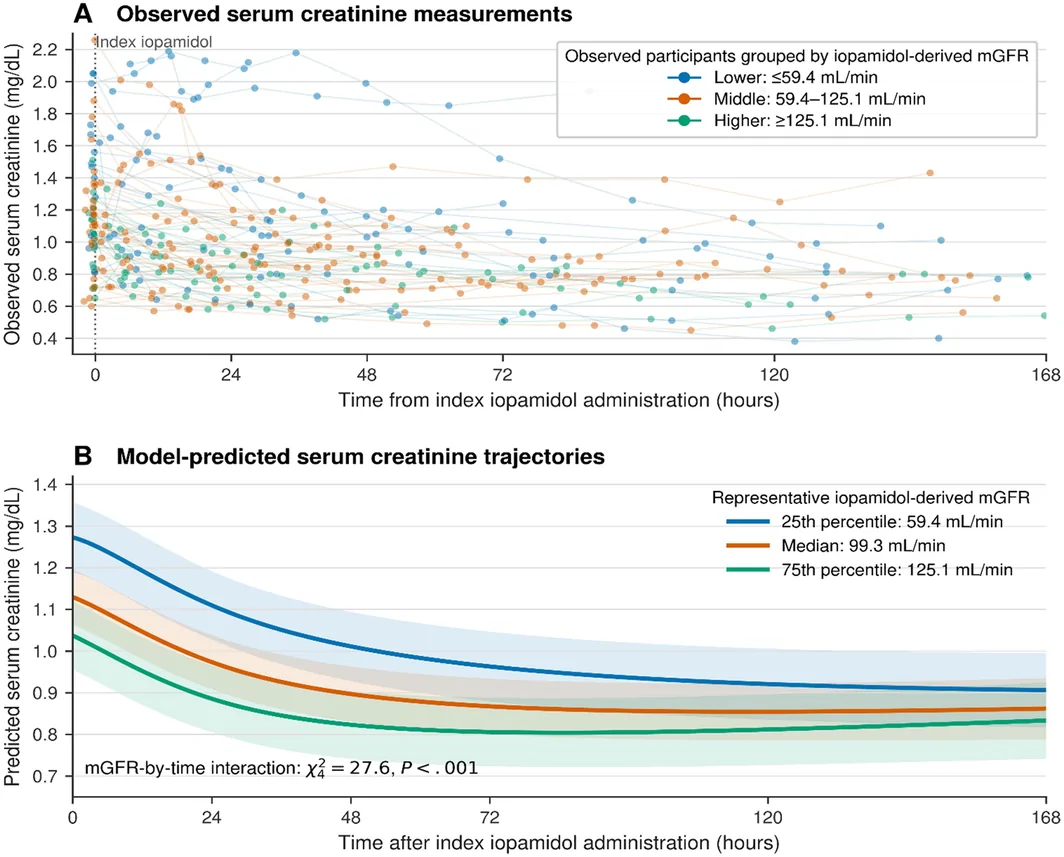
Observed and Model‐Predicted Serum Creatinine Trajectories according to iopamidol‐derived Measured Glomerular Filtration Rate (mGFR). (A) Observed serum creatinine (S_Cr_) measurements during the first 168 h after index iopamidol administration, including measurements obtained within 2 h before administration. Points represent individual observations, and faint lines connect repeated measurements from the same participant. Participants are grouped for visualization using the 25th and 75th percentiles of iopamidol‐derived measured glomerular filtration rate (mGFR). (B) Mixed‐effects model‐predicted S_Cr_ trajectories at the 25th percentile (59.4 mL/min), median (99.3 mL/min), and 75th percentile (125.1 mL/min) of mGFR. Shaded areas represent 95% confidence intervals. In the model, mGFR was analyzed as a continuous variable; the categories in panel A are used only to display the observed data. The overall mGFR‐by‐time interaction was significant (*p* < 0.001). The y‐axis ranges differ between panels to show the full range of observed values in panel A and the separation of predicted trajectories in panel B. Predicted trajectories are stratified by iopamidol‐derived mGFR measured at index imaging. Serial mGFR was not measured; therefore, the curves should not be interpreted as representing persistent differences in contemporaneous GFR throughout follow‐up. Using remnant serum after routine contrast‐enhanced trauma CT, opportunistic iopamidol measurement enabled cohort‐specific, model‐derived clearance estimates that were not reliably captured by creatinine‐based equations. With prospective validation, this approach could provide a timely kidney‐function anchor for dosing renally eliminated medications.

## 4 Discussion

Routine iopamidol administration during CE‐CT, combined with opportunistic sampling, provided an individualized measure of kidney filtration in this trauma cohort. Higher mGFR_IOP_ was associated with lower S_Cr_ near imaging, but this association attenuated during follow‐up. This exploratory finding illustrates the changing relationship between a filtration measurement obtained at trauma imaging and subsequent S_Cr_. It does not establish that participants maintained their index GFR throughout the following 7 days.

S_Cr_ is a circulating marker that requires time to accumulate or decline after GFR changes [3]. Creatinine‐based equations assume steady‐state S_Cr_, an assumption frequently violated during critical illness [3, 7, 8]. When GFR declines abruptly, S_Cr_ may remain deceptively low and overestimate current function; during recovery or augmented filtration, S_Cr_ may remain elevated and underestimate it. Similar S_Cr_ values can therefore represent different levels of kidney function depending on whether filtration is stable, declining, or recovering. A modeling‐based study estimated that S_Cr_ may require 12 to 72 h to meet AKI thresholds after a reduction in GFR [3]. Although this study was not designed to quantify that lag, the convergence of S_Cr_ trajectories illustrates how the relationship between measured filtration and S_Cr_ changes over time.

This delay has direct implications for medication dosing. More than half of patients with AKI at hospital admission in a single‐center study no longer met this AKI criteria by 48 h, suggesting that recovery often preceded its recognition by S_Cr_ [9]. Premature antibiotic dose reduction could therefore cause underexposure during a consequential treatment period. Conversely, rapidly declining GFR may not yet be reflected by S_Cr_, supporting doses that are too high.

Our findings extend another proof‐of‐concept study that repurposed iohexol administered during routine CE‐CT and residual blood samples to measure GFR in critically ill patients [10]. They found substantial individual disagreement with creatinine‐based estimates and detected worsening measured filtration approximately 2 days before its severity was apparent from eGFR [10]. Together, these proof‐of‐concept studies with iohexol and iopamidol help expand the application of this approach with CE‐CT because these two agents represent approximately 90% of the clinically used contrast agents.

eGFRcr was closer to iopamidol‐derived mGFR_IOP_ than CrCl overall, but neither was sufficiently accurate to be interchangeable with mGFR_IOP_. eGFR_cr_ had higher P30 and narrower limits of agreement but increasingly underestimated higher kidney function [8]. Failure to recognize high or augmented clearance may reduce exposure to antimicrobials and other kidney‐cleared medications [1, 9]. CrCl with actual body weight had less average bias, but its errors varied more widely among individuals, demonstrating that low average bias does not ensure accuracy for a particular patient [7]. These findings reinforce that acceptable population‐level performance does not ensure accuracy for individual dosing decisions [4]. Exogenous‐marker measurement provides patient‐specific information without relying exclusively on demographic characteristics and endogenous‐marker assumptions.

Renal‐dose recommendations are commonly linked to CrCl or eGFR_cr_ thresholds, yet differences among kidney‐function measures may place an individual patient on opposite sides of a dosing cutoff. Our findings do not establish which dose should be selected or demonstrate improved clinical outcomes. However, the wide individual errors and systematic underestimation by eGFR_cr_ at higher filtration rates identify circumstances in which creatinine‐based dosing may be unreliable. An opportunistic iopamidol‐derived filtration estimate obtained near trauma imaging could provide an additional anchor for dosing renally eliminated antimicrobials and other medications when kidney function is changing rapidly. Prospective studies should determine whether this information changes dose selection, pharmacokinetic target attainment, toxicity, or clinical outcomes.

Routine CE‐CT may therefore provide more than diagnostic imaging because a known contrast dose and limited blood sampling may reveal kidney filtration at a clinically important moment. This approach may be most useful when function is changing rapidly, augmented clearance is suspected, or an incorrect dose adjustment could compromise efficacy or safety. It could also complement, rather than replace, serial endogenous biomarkers by anchoring their subsequent trajectories to a direct filtration measurement. S_Cr_ remains valuable because it is readily available, but its trajectory should be interpreted as a delayed, multifactorial signal rather than an instantaneous measure of filtration.

Limitations include the retrospective design, sparse clinically timed sampling, reliance on population pharmacokinetic modeling, and absence of a measured pre‐trauma baseline, which is not practically obtainable in acute trauma care. Because the population model was re‐estimated in this study before individual clearance estimation, these findings represent cohort‐specific model adaptation rather than external validation of a transportable sparse‐sampling method. Prospective evaluation using fixed population parameters in an independent cohort is required to establish transportability. A single iopamidol‐derived mGFR does not establish the subsequent filtration trajectory or show that iopamidol caused changes in S_Cr_. These findings establish feasibility, not clinical interchangeability or improved outcomes. Injury severity, ICU status, bleeding, fluid administration, vasopressor exposure, and nephrotoxin use were not systematically collected and could have influenced subsequent S_Cr_. Because iopamidol clearance was measured only near imaging, we cannot determine whether narrowing S_Cr_ differences reflected changing filtration, resuscitation, altered creatinine generation, or other features of acute illness. Prospective validation with protocol‐defined sampling is needed to determine whether repurposing routine contrast exposure can provide timely, patient‐specific information that improves clinical decision‐making and outcomes.

## 5 Conclusions

Opportunistic measurement of iopamidol concentrations after routine CE‐CT may provide patient‐specific filtration estimates not reliably captured by S_Cr_ or creatinine‐based equations. Prospective validation is needed to determine whether this approach improves clinical decision‐making and patient outcomes.

## Author Contributions

**Manjunath P. Pai:** conceptualization, methodology, formal analysis, visualization, supervision, project administration, funding acquisition, writing – original draft, writing – review and editing. **Aleksas Matvekas:** investigation, data curation, project administration, writing – review and editing. **Lu Wang:** methodology, validation, investigation, resources, writing – review and editing. **Levi T. Hooper:** methodology, formal analysis, writing – review and editing. **June Sullivan:** methodology, resources, project administration, writing – review and editing. **Raymond Jean:** resources, writing – review and editing, investigation. **Kathleen A. Stringer:** writing – review and editing, investigation.

## Funding

Study activities were supported by departmental funds from the Department of Clinical Pharmacy, University of Michigan College of Pharmacy. KAS's effort was supported, in part, by the National Institutes of Health (NIH) through a National Institute of General Medical Sciences (NIGMS) R35GM136312. The content is solely the responsibility of the authors and does not necessarily represent the official views of NIH, NIGMS, or the University of Michigan.

## Conflicts of Interest

Kathleen Stringer is a Pharmacotherapy Scientific Editor and did not participate in editorial handling or review. Manjunath Pai is a member of the Pharmacotherapy editorial board. All authors declare that they have no conflicts of interest related to this work.

## Supporting information


**Figure S1:** Identification and selection of the TRACE study analytic cohort.
**Table S1:** Updated population pharmacokinetic model and individual‐parameter shrinkage.
**Table S2:** Agreement of creatinine‐based estimates with iopamidol‐derived mGFR.
**Table S3:** Sensitivity analyses according to pharmacokinetic sampling numbers and dose history.

## Data Availability

The data underlying this study are not publicly available due to institutional review board restrictions and the potential for patient re‐identification but are available from the corresponding author upon reasonable request and with appropriate institutional approvals.
