# Supplementary material for: Model‐Derived Iopamidol Clearance and Creatinine‐Based Kidney Function Estimates After Trauma: A Retrospective Cohort Study
Source: Pharmacotherapy. 2026 Nov 9;46(12):e70219. doi: 10.1002/phar.70219 (PMC13748343; doi:10.1002/phar.70219)
Supplement: Supplementary file 1 [file PHAR-46-0-s001.docx]

**Supporting Information**

**Model-Derived Iopamidol Clearance and Creatinine-Based Kidney Function Estimates After Trauma: A Retrospective Cohort Study**

*Supporting Information. Supplemental Methods and Supplemental Results*

Contents

[**Supplemental Methods**	2](#_Toc238028874)

[Study Design, Oversight, and Cohort Assembly	2](#_Toc238028875)

[Contrast Dose Reconstruction and Pharmacokinetic Data Quality Control	2](#_Toc238028876)

[Bioanalytical Measurement	3](#_Toc238028877)

[Population Pharmacokinetic Model	3](#_Toc238028878)

[Model Evaluation	3](#_Toc238028879)

[Creatinine-Based Kidney-Function Estimates	4](#_Toc238028880)

[Agreement Analyses	4](#_Toc238028881)

[Longitudinal Serum Creatinine Analysis	4](#_Toc238028882)

[Sensitivity Analyses	4](#_Toc238028883)

[**Supplemental** **Results**	5](#_Toc238028884)

[Cohort, Exposure, and Sampling	5](#_Toc238028885)

[Population Model and Individual Clearance	5](#_Toc238028886)

[Agreement With Creatinine-Based Estimates	5](#_Toc238028887)

[Serum Creatinine Trajectories	6](#_Toc238028888)

[Sensitivity Analyses	6](#_Toc238028889)

[**Supplemental** **Tables**	7](#_Toc238028890)

[Figure S1. Identification and selection of the TRACE study analytic cohort.	7](#_Toc238028891)

[Table S1. Updated Population Pharmacokinetic Model and Individual-Parameter Shrinkage	8](#_Toc238028892)

[Table S2. Agreement of Creatinine-Based Estimates With Iopamidol-Derived mGFR	9](#_Toc238028893)

[Table S3. Sensitivity Analyses According to Pharmacokinetic Sampling Numbers and Dose History	9](#_Toc238028894)

[Abbreviations	9](#_Toc238028895)

# Supplemental Methods

## Study Design, Oversight, and Cohort Assembly

The Trauma Renal Function Assessment by Contrast Enhancement (TRACE) study was a single-center retrospective observational cohort study conducted at the University of Michigan. The University of Michigan Medical School Institutional Review Board approved the study under expedited category 5 with waivers of informed consent and HIPAA authorization (HUM00275396). This feasibility pilot planned to include a convenience sample of 60–90 patients. Potentially eligible patients were identified from a trauma case log generated in the electronic health record and reviewed by study personnel twice weekly between September 2025 and March 2026. The log was screened for adults evaluated for traumatic injury who had undergone iopamidol-enhanced computed tomography. For potentially eligible patients, requests for available remnant serum specimens were submitted to Clinical Pathology after completion of clinical testing. Because cases were identified during twice-weekly reviews and inclusion depended on the availability of suitable remnant specimens, patients were not identified or included sequentially.

Patients were eligible for specimen retrieval if they were aged 18 years or older, had undergone contrast-enhanced computed tomography, and had at least three remnant clinical specimens collected within 72 hours of imaging. The analytic cohort required at least one quantifiable postcontrast iopamidol concentration, a participant-specific conditional-mode clearance estimate, and at least one serum creatinine (SCr) measurement.

Demographic characteristics, height, weight, contrast formulation, administered volume, exact contrast administration time, clinical laboratory results, and repeat contrast administrations were abstracted from the electronic health record. No unobserved pretrauma SCr value was imputed. The index event was the first verified iopamidol administration; all subsequent verified contrast administrations were retained as separate dose events in the pharmacokinetic history**.**

## Contrast Dose Reconstruction and Pharmacokinetic Data Quality Control

Administered iopamidol mass was reconstructed from the documented product and volume. Concentrations of 612 mg/mL and 755 mg/mL were used for Isovue-300 and Isovue-370, respectively. The final pharmacokinetic file contained 109 verified dose events and 177 measured concentrations. Exact clock times were converted to elapsed hours from the index dose, and time after the most recent dose was retained for descriptive summaries. Brief power-injector administrations were represented as intravenous boluses.

Dose rows duplicated in the source extract were removed after comparison of administration time, formulation, and volume. Same-time concentration replicates were retained as separate observations because each represented an independently assayed remnant specimen. One detectable predose value was excluded from the likelihood (missing dependent variable indicator) but retained in the quality-control record. One result below the lower limit of quantification was handled as a left-censored observation at 0.1 mg/L. Accordingly, 176 postcontrast concentrations contributed to model estimation.

## Bioanalytical Measurement

Remnant serum specimens were stored at -70 C until analysis. Iopamidol concentrations were quantified by the previously described liquid chromatography-mass spectrometry method. In brief, protein precipitation was performed with methanol containing iohexol-d5 as internal standard, followed by chromatographic separation on a polar C18 column and positive-ion multiple-reaction monitoring. Calibration standards covered 0.1 to 500 micrograms/mL, with dilution of samples above the calibration range and low-, mid-, and high-concentration quality-control samples included across analytical runs.

## Population Pharmacokinetic Model

Pharmacokinetic analyses were performed in MonolixSuite 2024R1 (Lixoft SAS, Antony, France). The previously developed covariate-free iopamidol model supplied the structural framework and informative starting distributions. The model comprised two disposition compartments, first-order elimination from the central compartment, and the parameters clearance (CL), central volume (V1), intercompartmental clearance (Q), and peripheral volume (V2). Interindividual variability was modeled exponentially for CL, V1, Q, and V2, with log-normal individual parameter distributions. No correlations among random effects were included. Residual unexplained variability was described by a combined additive and proportional error model.

The population fixed effects, random-effect distributions, and residual-error parameters were updated using the stochastic approximation expectation-maximization (SAEM) algorithm. Published base-model estimates informed initialization and prior distributions but were not held fixed. This strategy allowed the population distribution to adapt to the markedly higher contrast doses, irregular sampling times, repeated administrations, and clinical heterogeneity represented in TRACE. S_Cr_, Cockcroft-Gault creatinine clearance (CrCl), and estimated GFR (eGFR) were deliberately excluded as clearance covariates so that the creatinine-based comparators were not mathematically incorporated into the iopamidol clearance estimate.

After population estimation, the conditional distribution of individual parameters was calculated for each participant. The mode of this conditional distribution was used as the participant-specific empirical Bayes, or maximum a posteriori, estimate. Individual CL was converted from L/h to mL/min by multiplying by 1000/60 and was interpreted as absolute, nonindexed iopamidol-derived measured GFR (mGFR). Individual V1, Q, and V2 estimates were not interpreted clinically because opportunistic samples were concentrated in the postdistribution and terminal phases.

## Model Evaluation

SAEM convergence was assessed from parameter and objective-function trajectories. Model adequacy was evaluated using observed-versus-population and observed-versus-individual predictions, individual concentration-time fits, individual weighted residuals, normalized prediction distribution errors, random-effect distributions, and a visual predictive check. Parameter precision was summarized by stochastic-approximation standard errors, relative standard errors, and approximate 95% confidence intervals. Conditional-mode shrinkage was examined separately for each individual parameter; CL was the prespecified individualized end point.

## Creatinine-Based Kidney-Function Estimates

Cockcroft-Gault CrCl was calculated from the first available S_Cr_, age, sex, and recorded body weight. In addition, we also estimated the alternate weight descriptors of ideal body weight, adjusted body weight, and lean body weight as a substitute for recorded body weight. Given that a combination of actual weight, ideal body weight, and adjusted body weight is sometimes used in practice to a dosing weight, this was also computed. Dosing weight used actual weight if less than ideal body weight, adjusted body weight if ≥1.25 x ideal body weight, or ideal body weight if neither condition was met. The 2021 CKD-EPI creatinine eGFR was calculated in mL/min/1.73 m^2^ and converted to absolute units for direct comparison with iopamidol clearance by multiplying by body surface area/1.73. Body surface area was calculated by the Mosteller equation. Iopamidol-derived mGFR, Cockcroft-Gault CrCl, and nonindexed CKD-EPI eGFR were therefore compared on the same mL/min scale.

## Agreement Analyses

For each creatinine-based estimator, the difference was defined as the estimator minus iopamidol-derived mGFR. Agreement was summarized using mean bias, standard deviation of the differences, mean and median absolute error, root mean squared error, the proportions within 20% and 30% of mGFR (P20 and P30), and Bland-Altman 95% limits of agreement (mean bias +/- 1.96 standard deviations). Proportional bias was evaluated by regressing the difference on the pairwise mean. Paired P30 values were compared using an exact McNemar test. Correlation was considered descriptive and was not interpreted as evidence of agreement.

## Longitudinal Serum Creatinine Analysis

All available S_Cr_ measurements were aligned to time from the index iopamidol administration. The longitudinal dataset contained 449 measurements from 76 participants (median, 5.5; interquartile range [IQR], 3-7 per participant). S_Cr_ was modeled using a linear mixed-effects model with a participant-specific random intercept. Time from the index dose was represented by a cubic spline with 4 degrees of freedom. Iopamidol-derived mGFR was centered and scaled so that its main effect represented a 30-mL/min difference at time 0. Interactions between mGFR and all spline terms allowed the association between mGFR and S_Cr_ to vary over time. The overall interaction was evaluated by a likelihood-ratio test comparing models with and without the mGFR-by-time interaction terms.

Predicted S_Cr_ trajectories and 95% confidence intervals were generated at the 25th percentile, median, and 75th percentile of mGFR. Figure 2 displays the clinically relevant window from 2 hours before through 168 hours after iopamidol administration and contains 420 of the 449 available S_Cr_ measurements. The mGFR groups shown with observed data were defined only for visualization; mGFR remained continuous in the mixed-effects model. Missing S_Cr_ values were not imputed.

## Sensitivity Analyses

Because participant-specific estimates can be influenced by sampling number, agreement analyses were repeated among participants with at least 2 and at least 3 modeled iopamidol concentrations. A separate analysis was restricted to participants with a single verified contrast administration. These analyses assessed whether the direction of the comparison between CKD-EPI, Cockcroft-Gault, and iopamidol-derived mGFR was dependent on the sparsest pharmacokinetic profiles or repeat-dose histories.

# Supplemental Results

## Cohort, Exposure, and Sampling

Of 90 trauma cases reviewed in the electronic health record, 84 met the eligibility criteria and had potentially available remnant specimens. Remnant specimens were retrievable for 80 patients. Four patients were excluded because none of their retrieved specimens yielded at least one quantifiable iopamidol concentration, leaving a final analytic cohort of 76 participants (Figure S1).

Median age was 60 years (IQR, 44–74 years), 48 participants (63.2%) were male, and median body mass index was 28.8 kg/m² (IQR, 24.8–32.5 kg/m²) (Table 1). The first available indexed CKD-EPI 2021 eGFR was <60 mL/min/1.73 m² in 26 participants (34.2%) and ≥90 mL/min/1.73 m² in 19 (25.0%). The median initial iopamidol dose was 76.5 g (IQR, 75.4–76.5 g).

The 176 modeled concentrations corresponded to specimens collected a median of 18.6 hours (IQR, 8.1–30.4 hours) after the most recent iopamidol dose, with a range of 0.72–109.4 hours. Participants contributed a median of 2 concentrations (IQR, 1–3): 25 contributed 1 concentration, 22 contributed 2, 17 contributed 3, 5 contributed 4, 6 contributed 5, and 1 contributed 6. Overall, 51 participants had at least 2 concentrations and 29 had at least 3. Fifty participants had a single verified dose event, 20 had 2 dose events, 5 had 3, and 1 had 4..

## Population Model and Individual Clearance

The SAEM algorithm converged, and the updated typical CL was 4.942 L/h (82.4 mL/min; relative standard error, 8.7%) (Table S1). Interindividual variability in CL was 75.4% coefficient of variation, reflecting the broad kidney-function range in the trauma cohort. Conditional-mode shrinkage was 11.9% for CL, compared with 71.2% to 86.5% for V1, Q, and V2. Accordingly, only individual CL was carried forward as the patient-specific filtration phenotype.

Observed concentrations were generally aligned with individual predictions across the wide concentration range. Residual and prediction-distribution diagnostics supported use of the model for individual CL estimation, while the high shrinkage of distributional parameters indicated that the sparse clinical sampling provided limited participant-level information about V1, Q, and V2. Median iopamidol-derived mGFR was 99 mL/min (IQR, 59-125 mL/min).

## Agreement With Creatinine-Based Estimates

Complete agreement results are shown in Table S2. Cockcroft-Gault CrCl had a mean bias of -4.0 mL/min but wide 95% limits of agreement (-82 to 74 mL/min), a root mean squared error of 40 mL/min, and P30 of 59%. Nonindexed CKD-EPI eGFR had a more negative mean bias (-7.7 mL/min) but narrower limits of agreement (-73 to 57 mL/min), a lower root mean squared error (34 mL/min), and higher P30 (71%). CKD-EPI was within 30% of mGFR for 12 participants in whom Cockcroft-Gault was not, whereas the opposite occurred for 3 participants (exact McNemar P=.035). When Cockcroft-Gault CrCl was calculated using ideal, adjusted, dosing, and lean body weight, P30 was 39%, 58%, 54%, and 29%, respectively, compared with 59% using actual body weight.

Cockcroft-Gault error did not vary systematically across the measured range (P=.862 for proportional bias). In contrast, CKD-EPI increasingly underestimated mGFR as the pairwise mean increased (P<.001), demonstrating that its better overall precision did not eliminate systematic error at higher kidney function. Cockcroft-Gault calculations using ideal, adjusted, dosing, and lean body weight also increasingly underestimated mGFR as the pairwise mean increased (all P≤.009).

## Serum Creatinine Trajectories

At the time of index iopamidol administration, each 30-mL/min higher mGFR was associated with a 0.11-mg/dL lower S_Cr_ (95% CI, 0.06-0.16 mg/dL lower; P<.001). The overall mGFR-by-time interaction was significant (likelihood-ratio P<.001), indicating that the relationship between the directly measured filtration phenotype and S_Cr_ changed during follow-up. Model-predicted S_Cr_ trajectories at mGFR values of 59, 99, and 125 mL/min became progressively more similar over 7 days despite their clear separation near the index CT examination.

## Sensitivity Analyses

The principal comparison was unchanged after restriction by pharmacokinetic sampling depth (Table S3). Among participants with at least 2 concentrations, P30 was 69% for CKD-EPI and 57% for Cockcroft-Gault; among those with at least 3 concentrations, the corresponding values were 62% and 55%. In both subsets, CKD-EPI retained narrower limits of agreement and lower root mean squared error. Among the 50 participants with a single verified dose event, CKD-EPI P30 was 70% compared with 64% for Cockcroft-Gault, and its limits of agreement remained narrower. Differences in the absolute percentages across subsets reflect changes in case mix and mGFR distribution and should not be interpreted as a within-person comparison of sampling strategies.

# Supplemental Tables

# Figure S1. Identification and selection of the TRACE study analytic cohort.

Potential cases were identified through twice-weekly review of an EHR trauma case log. This retrospective feasibility study used a convenience sample and did not involve prospective or sequential enrollment. EHR, electronic health record; TRACE, Trauma Renal Function Assessment by Contrast Enhancement.

# Table S1. Updated Population Pharmacokinetic Model and Individual-Parameter Shrinkage

| Parameter | Estimate | SE | RSE, % | Approximate 95% CI | Shrinkage, % |
| --- | --- | --- | --- | --- | --- |
| Typical CL, L/h | 4.942 | 0.428 | 8.65 | 4.175-5.850 | 11.9 |
| Typical V1, L | 29.015 | 2.103 | 7.25 | 25.186-33.426 | 71.2 |
| Typical Q, L/h | 0.278 | 0.0393 | 14.13 | 0.212-0.366 | 86.5 |
| Typical V2, L | 3.762 | 0.327 | 8.70 | 3.175-4.457 | 85.2 |
| Omega CL | 0.671 | 0.0685 | 10.21 | 0.550-0.818 | - |
| Omega V1 | 0.238 | 0.157 | 65.97 | 0.0839-0.677 | - |
| Omega Q | 0.272 | 0.0861 | 31.66 | 0.152-0.486 | - |
| Omega V2 | 0.232 | 0.0787 | 33.93 | 0.125-0.430 | - |
| Additive error a, mg/L | 0.134 | 0.0452 | 33.68 | 0.0727-0.248 | - |
| Proportional error b | 0.352 | 0.0297 | 8.45 | 0.298-0.415 | - |

Typical CL of 4.942 L/h is equivalent to 82 mL/min. Estimated random-effect standard deviations corresponded to coefficients of variation of 75.4%, 24.2%, 27.7%, and 23.5% for CL, V1, Q, and V2, respectively. Shrinkage is reported for the conditional-mode individual parameters. Abbreviations: CI, confidence interval; CL, clearance; Q, intercompartmental clearance; RSE, relative standard error; SE, standard error; V1, central volume; V2, peripheral volume.

**Omega** describes how much a pharmacokinetic parameter varies between patients after accounting for measured characteristics such as kidney function, weight, or age. A larger omega means greater unexplained patient-to-patient variability.

**Shrinkage** describes how strongly an individual patient’s estimated parameter is pulled toward the population average because that patient’s concentration data provide limited information. Low shrinkage means the individual estimates are well informed by the observed concentrations. High shrinkage means they largely reflect the population model and should not be interpreted as precise patient-specific values.

**Interpretation**: Because clearance was the prespecified individualized end point, its low conditional-mode shrinkage (11.9%) indicates that participant-specific clearance estimates were informed by the observed concentrations rather than being strongly pulled toward the population mean. In contrast, the clearance omega of 0.671, corresponding to a coefficient of variation of 75.4%, demonstrates substantial between-participant variability in clearance, consistent with the broad range of kidney function in this cohort.

# Table S2. Agreement of Creatinine-Based Estimates With Iopamidol-Derived mGFR

| Estimator | Bias | SD | MAE | RMSE | P20, % | P30, % | 95% limits |
| --- | --- | --- | --- | --- | --- | --- | --- |
| CKD-EPI eGFRcr | -7.7 | 33.1 | 25.1 | 33.8 | 47.4 | 71.1 | -72.6 to 57.2 |
| CG CrCl_wt | -4.0 | 39.5 | 28.6 | 39.5 | 39.5 | 59.2 | -81.5 to 73.5 |
| CG CrCl_ibw | −26.1 | 31.6 | 31.8 | 40.9 | 22.4 | 39.5 | −88.1 to 35.9 |
| CG CrCl_adjbw | −17.1 | 33.1 | 28.4 | 37.1 | 34.2 | 57.9 | −82.1 to 47.8 |
| CG CrCl_dw | −19.1 | 33.1 | 29.3 | 38.0 | 27.6 | 53.9 | −83.9 to 45.8 |
| CG CrCl_lbw | −31.5 | 32.6 | 35.7 | 45.1 | 18.4 | 28.9 | −95.3 to 32.4 |

All error quantities are in mL/min. Difference = creatinine-based estimate minus mGFR. The exact paired comparison of P30 values yielded P=.035. Proportional-bias P values were .862 for Cockcroft-Gault and <.001 for CKD-EPI. Abbreviations: CKD-EPI, Chronic Kidney Disease Epidemiology Collaboration; CG CrCl, Cockcroft-Gault creatinine clearance based on different weight; _wt, actual body weight; _ibw, ideal body weight; _adjbw, adjusted body weight; _dw, dosing weight, _lbw, lean body weight; MAE, mean absolute error; mGFR, measured glomerular filtration rate; P20/P30, proportion within 20%/30% of mGFR; RMSE, root mean squared error; SD, standard deviation of the differences.

# Table S3. Sensitivity Analyses According to Pharmacokinetic Sampling Numbers and Dose History

| Analysis Cohort | N | mGFR, median [IQR] | CG P30, % | CKD-EPI P30, % | CG RMSE | CKD-EPI RMSE |
| --- | --- | --- | --- | --- | --- | --- |
| All participants | 76 | 99.3 [59.4-125.1] | 59.2 | 71.1 | 39.5 | 33.8 |
| >=2 concentrations | 51 | 87.8 [50.7-123.9] | 56.9 | 68.6 | 36.2 | 29.4 |
| >=3 concentrations | 29 | 87.8 [46.5-130.7] | 55.2 | 62.1 | 35.1 | 31.6 |
| Single-dose participants | 50 | 94.8 [60.3-121.3] | 64.0 | 70.0 | 35.0 | 30.3 |

RMSE values are in mL/min. Subset comparisons are descriptive because sampling depth and repeat-dose status were determined by clinical care and were not randomized. Abbreviations: CG, Cockcroft-Gault; CKD-EPI, Chronic Kidney Disease Epidemiology Collaboration; IQR, interquartile range; mGFR, measured glomerular filtration rate; P30, proportion within 30% of mGFR; RMSE, root mean squared error.

# Abbreviations

AIC, Akaike information criterion; BIC, Bayesian information criterion; BSA, body surface area; CKD-EPI, Chronic Kidney Disease Epidemiology Collaboration; CL, clearance; CrCl, creatinine clearance; eGFR, estimated glomerular filtration rate; IIV, interindividual variability; IQR, interquartile range; LC-MS, liquid chromatography-mass spectrometry; MAP, maximum a posteriori; MAE, mean absolute error; mGFR, measured glomerular filtration rate; P20/P30, proportion within 20%/30% of mGFR; Q, intercompartmental clearance; RMSE, root mean squared error; RSE, relative standard error; SAEM, stochastic approximation expectation-maximization; S_Cr_, serum creatinine; V1, central volume; V2, peripheral volume.
